# Supplementary material for: Excessive phosphorus loading contributes to future vulnerability of mangrove ecosystems by reducing net ecosystem exchange of carbon
Source: Camb Prism Coast Futur. 2026 Apr 1;4:e6. doi: 10.1017/cft.2026.10025 (PMC13122390; doi:10.1017/cft.2026.10025)
Supplement: Krauss et al. supplementary material [file S2754720526100250sup001.pdf]

Cambridge Prisms: Coastal Futures

Supplementary material

www.cambridge.org/cft

Excessive phosphorus loading contributes to future vulnerability of mangrove ecosystems by reducing net ecosystem exchange of carbon

Ken W. Krauss, Jeremy R. Conrad, Jamie A. Duberstein, Eric J. Ward, Judith Z. Drexler, Kevin J. Buffington, Brian W. Benscoter, Haley Miller, Natalie T. Faron, Sergio L. Merino, Andrew S. From, Elista Peneva-Reed, Zhiliang Zhu, Karen M. Thorne and Ilka C. Feller*

** Affiliations listed in the main document*

Research Paper

Supplement to: Krauss KW, Conrad JR, Duberstein JA, Buffington KJ, Benscoter BW, Miller H, et al. (2025). Excessive phosphorus loading contributes to future vulnerability of mangrove ecosystems by reducing net ecosystem exchange of carbon. *Coastal Futures*

Received: 24 July 2025

Corresponding author:
Ken W. Krauss;
Email: kkrauss@lumcon.edu

© The Author(s), 2025. Published by Cambridge University Press. This is an Open Access article, distributed under the terms of the Creative Commons Attribution license (http://creativecommons.org/licenses/by/4.0), which permits unrestricted re-use, distribution and reproduction, provided the original article is properly cited.

Expanded introduction

Hydrologic disturbance

Mangroves are tolerant to various amounts of environmental stress, but eventually succumb to hydrologic change when near-permanent inundation or enhanced soil anaerobiosis result. Soil compaction through reduced root turnover (Krauss et al. 2018) and stress to invertebrate community oxygenation (Demopoulos et al. 2024) create a vulnerability to future acute stress events, resulting in habitat collapse (Lewis et al. 2016; Chambers et al. 2019). The time between the original hydrologic modification and mortality often takes decades, and by that time, cause of mangrove collapse is sometimes attributed to an unrelated event, such as a tropical cyclone (Krauss et al. 2023), offering little opportunity for timely remediation. Furthermore, hydrologic alteration and nutrient condition are often correlative, making site selection of mangroves with limited hydrologic alteration critical for understanding fertilizer-specific effects.

Nutrient loading

Phosphorus (P) enrichment facilitated greater decomposition of belowground roots and greater P-resorption efficiency in Belize, while nitrogen (N)-resorption was stimulated by P-enrichment only in long-hydroperiod stunted mangroves (Feller et al. 2002). Nutrient limitation, from N to P, changed along that same hydroperiod gradient in the Belizean mangrove forest, but a sub-tropical mangrove forest responded with consistent N limitation despite hydroperiod shifts (Feller et al. 2003). Stress by eutrophication appears unsurprisingly variable depending on the mangrove system, and perhaps the antecedent nutrient biogeochemistry for which the forest has become accustomed during development or from subsequent modifications. For a mangrove forest in Puerto Rico, soil P amendment did not stimulate greater leaf decomposition or carbon mineralization (Jessen et al. 2021) as it did on other Caribbean sites (Lovelock et al. 2014b); however, the addition of P contributed to soil surface elevation loss in some mangrove sites in southwest Florida (Conrad et al. 2024). Greater P loading may increase shoot vs. root growth, leading to reduced shear strength of sediments and greater erosive loss (Lovelock et al. 2009).

Expanded methods

Site description

Mangrove forests on Sanibel Island are protected from further development as part of the National Wildlife Refuge System (as J.N. "Ding" Darling National Wildlife Refuge, or DDNWR). DDNWR is approximately 2,571 ha in size, consisting of aquatic habitat, beach strand vegetation, and mangrove forest. Sanibel Island is subjected to the natural environmental threats of hurricanes (Meyers et al. 2006; Milbrandt et al. 2006) and sea-level rise. When healthy, mangrove forests are arguably the most resilient of Sanibel's natural habitats to both threats. Healthy mangroves often recover expediently from tropical cyclones (Krauss and Osland 2020), even though they look ragged for years to follow, and mangroves have inherent mechanisms to cope with rising sea levels (McKee et al. 2007; Krauss et al. 2014b), as long as relative sea-level rise is not too extreme (Saintilan et al. 2020). Furthermore, Sanibel Island has all of the common neotropical mangrove species that occur in Florida and the Caribbean region, including *Rhizophora mangle*, *Avicennia germinans*, and *Laguncularia racemosa*. *Conocarpus erectus*, a mangrove associate, even occupies slightly higher elevations along land boundaries, roads, and spoil banks. This diversity of mangrove types provides growth characteristics that incorporate morphological flexibility in the response of the mangroves to different environmental stressors (Snedaker 1995).

Tests of fertilizer treatment efficacy

Delivery of fertilizers to coastal wetlands for experimental study is predicated on two things: (1) persistence over the period assumed, and (2) dispersal within the rhizosphere of all trees in a plot. We tested the efficacy of using granular inorganic nutrients specifically on Sanibel Island through repetitive porewater sampling after fertilizer application (Miller 2022). Porewater sampling was conducted three-days prior to nutrient application using sipper tubes to extract water at 15 cm soil depths (N=36/per sampling). Sampling was repeated spatially at 0.5 m, 1.0 m, and 1.5 m from nutrient applications at cardinal directions. Sampling occurred weekly for a duration of two months following fertilization, bi-weekly for the next two months, and monthly for the final two months of the study (Miller 2022). Each sample was measured for nitrate [NO_3^-], ammonia [NH_3^+], and phosphate [PO_4^-] using a SYSTEA Easychem Plus Analyzer (Anagni, Italy). We discovered that our N and P fertilization methods were sufficient and effective for fertilizing a radius of at least 0.5 m over a 6-month interval, which would overlap our 16 grid points. We did not find significant spatial effect of N fertilization in soil porewater NH_3^+ or NO_3^- concentrations, and temporal effects showed very slow rates of inorganic soil N buildup over the course of our study. Indeed, we did find statistically significant fixed effects of time on [PO_4^-], [NO_3^-], and [NH_3^+]. However, none of our results showed negative relationships of individual porewater inorganic nutrient concentrations with time and, thus no drawdown effect over time was detected. Thus, our application supports past results in that experimental fertilizers used on Sanibel Island reached rhizospheres and persisted (Feller 1995; McKee et al. 2002; Feller et al. 2003; McKee et al. 2007).

Sap flow instrumentation

We measured stem water flux at various radial depths into trees growing on N-fertilized plots (+N), P-fertilized plots (+P), and control plots using sap flow techniques. Sap flow techniques use a variety of approaches that can relate water use characteristics to ecosystem stress (Smith and Allen 1996; Krauss et al. 2007); we chose to use thermal dissipation probes (TDP). Sap flow studies were initiated one year after fertilization with probes connected via cables to voltage regulators and data loggers (model CR1000, Campbell Scientific, Logan, UT, USA). Our TDP systems were configured commercially (model FLGS-TDP, Dynamax, Inc., Houston, TX, USA), and each consisted of 20 wires connected to 32 TDP pairs for *d*T determinations (see Granier 1987 for theory). We deployed three FLGS-TDP systems. The 32 *d*T pairs were

pre-configured to measure sapwood depths of 5 mm (8-12 *d*T sensors), 15 mm (8-12 *d*T sensors), 50 mm (4 *d*T sensors), 70 mm (4 *d*T sensors), and 90 mm (4 *d*T sensors). Because we hypothesized that changes in sap flow would be most evident at the sapwood depths formed during fertilization, we prioritized 5 mm and 15 mm depths for measurement. Growth of DDNWR's mangrove trees ranged from 0.8 to 1.2 cm^2 y^{-1} of basal area during fertilization periods (Conrad 2022), corresponding to those focal depths.

Weather station instrumentation

A partial weather station was deployed approximately 5 km from sap flow studies, and recorded air temperature (°C), relative humidity (%), photosynthetic photon flux density (μmol m^{-2} s^{-1}), barometric pressure (MPa), and rainfall (mm). A second weather station, maintained by DDNWR staff on Sanibel Island was used as a source of radiation data (W m^{-2}), and supplemental air temperature and rainfall data. These stations were used to determine vapor pressure deficit (D, kPa); D and sap flow are strongly correlated (Bovard et al. 2005).

Soil (R_s) and pneumatophore (R_p) CO_2 flux instrumentation

Soil CO_2 fluxes (R_s) were measured using a portable infrared gas analyzer (model Li-8100a, Li-Cor Environmental, Inc., Lincoln, NE, USA). Chambers for R_s were 20-cm diameter (or 314 cm^2) and were devoid of aerial roots during in-situ measurements over 4 days each in June (wet season) and November (dry season). Control, +N, and +P treatments had 10, 4, and 4 collars, respectively, for a total of 18 collars, spaced at least 0.7-m apart. Collars were inserted to a depth of 0.5 cm and were allowed to sit for at least 30 minutes prior to sampling. Soil temperature and moisture were measured during R_s measurements with probes (models Omega soil T probe and Delta-T soil moisture probe, Li-Cor Environmental, Inc., Lincoln, NE, USA).

CO_2 fluxes through pneumatophores (R_p) were measured in-situ using a different type of infrared gas analyzer (model EGM-4, PP Systems, Inc., Amesbury, MA, USA). The gas analyzer was attached by Nalgene tubing to gas ports on a 1.3-cm diameter PVC chamber. The chamber was fit over an individual pneumatophore, inserted in the soil at the base of the pneumatophore, and sealed with a latex skirt on top to isolate pneumatophore tissue response. Volume and surface area of the pneumatophore were determined, and R_p adjusted to ground area (Faron 2021). Three pneumatophores were sampled from each treatment combination coincident with the timing of R_s measurements. Data for R_s and R_p are available in Benscoter and Faron (2023).

Table S1 Linear regression parameter estimates for predicting soil (R_s) and pneumatophore (R_p) fluxes of CO_2 from soil/air temperature (x) for nitrogen (+N), phosphorus (+P), and control treatments at Ding Darling NWR as $y = mx + b$ (Faron 2022)

	g CO_2 m^{-2} y^{-1}	
Parameter	y (R_s)	y (R_p)
**+N**		
m	0.0120	--
b	-0.14	--
r^2	0.453	--
$P > F$	< 0.001	0.999
**+P**		
m	0.0014	0.0330
b	0.15	-0.64
r^2	0.090	0.385
$P > F$	0.0190	0.0030
**Control**		
m	-0.0014	0.0590
b	0.20	-1.30
r^2	0.082	0.764
$P > F$	0.0210	0.0050

Soil surface carbon accumulation (SSCA)

Marker horizons of powdered feldspar clay were laid to determine rates of vertical accretion, and those data were converted to SSCA for control, +N, and +P plots. This approach assumes that the C content of the sediments and fine root in-growth

matches upper horizon organic C content reported by Drexler (2019). SSCA determination by this approach has precedent (Lovelock et al. 2014a; Noe et al. 2016; Cormier et al. 2022; see Dahl et al. 2025).

Gross primary productivity, net primary productivity, and net ecosystem exchange

We determined gross primary productivity (GPP) using species-specific leaf-scale instantaneous water use efficiencies (WUE_i) developed separately for control, +N, and +P treatments. Leaf-scale WUE_i can scale reasonably well from leaf-to-canopy (Linderson et al. 2012; Liang et al. 2022), and we use this scaling here to provide a molar accounting of CO_2 taken up per unit water used by the forest though our STrAP model. However, it is important to recognize that WUE_i is sensitive to large variations in D; WUE_i scales theoretically as $1/1.6D$ (Liang et al. 2023). Given that D was 0.48 ± 0.4 kPa (S.D.) (1.5 years; at 15-min intervals) at DDNWR, we held WUE_i constant to compare among control, +N, and +P, making the experimental assumption that all treatments held to the scaler of $1/1.6D$ and that changes in WUE_i with D likely occur but are consistent across treatment. Canopy respiration (R_c) was subtracted from GPP to yield net primary productivity (NPP). Along with NPP, soil respiration (R_s) and pneumatophore respiration (R_p) were accounted when determining net ecosystem exchange (NEE) of C. Equations for determining R_s and R_p using soil/air temperature are reported in Table S1. Closure analysis is described in Box 1.

Water use efficiency (WUE_i) and its usage

Different WUE_i conversions were developed using an infrared gas analyzer (model Li-6800, Li-Cor Environmental, Inc., Lincoln, NE, USA) by species and treatment for *A. germinans* and *R. mangle* from DDNWR and derived as a single value across treatments for *L. racemosa* (Krauss 2004; Pezeshki et al. 1990) (Table S2). WUE_i data were combined with stand water use and transformed to a CO_2 canopy uptake rate and converted from molar units to mass (g CO_2 m^{-2} h^{-1}), and then to C values (g C m^{-2} h^{-1}), producing our estimates of GPP. Once adjusted to NPP, NEE was determined by subtracting R_s and R_p. Coarse woody debris respiration was not included in R_s values; however, we also made no adjustment for basal area or downed wood occupation of soils, which would partially compensate. Open ground soil flux differentials would not be large relative to fluxes from woody debris (Troxler et al. 2015). Methane fluxes were not included but are generally very low from wetlands with salinity > 18 psu (Poffenbarger et al. 2011; Holm et al. 2016). Mean salinity from plots near open water and more interior at DDNWR was 35 and 46, respectively (Conrad et al. 2024).

Table S2 Instantaneous photosynthetic water use efficiency (WUE_i) values used for converting stand water use to Gross Primary Productivity (GPP) by nitrogen (+N), phosphorus (+P), and control simulations at Ding Darling NWR

	WUE_i, μmol CO_2 (mmol H_2O)$^{-1}$		
Species	+N	+P	Control
Rhizophora mangle	8.54	3.74	7.37
Avicennia germinans	6.21	6.74	8.04
Laguncularia racemosa	4.03	4.03	4.03

Expanded discussion

Plot-scale eco-physiological changes

Since new basal area growth stimulated by nutrient addition would likely be reflected in our shallowest sap flow (J_s) measurements of 5 mm, and because N and P availability impacts water uptake and transport in plants (Carvajal et al. 1996; Clarkson et al. 2000), we expected to see the largest increase in J_s with +N treatment and +P treatment at radial sapwood depths of 5 mm. We did not, despite different WUE_i and treatment influences on individual tree water use. However, we did find larger variability in J_s among +P-treated *A. germinans* at 5 cm and 15 cm radial depths. Generally, however, it appears that water transport within the most responsive tissue is not likely being limited by nutrients, or

even affected by nutrients, among most trees. Based on high R_s, mineralization of soil N and P appear adequate to support mangrove water uptake (or flux). For +P, the duration of higher R_s and R_p sustainment is not known but may reach a tipping point in time as greater submergence to sea-level rise occurs (e.g., Wang et el. 2023). *Avicennia germinans* trees do appear to be operating at their eco-physiological maximum with the nutrient concentrations present at DDNWR, but this might not be the case for *R. mangle*. Past study of *A. germinans* in Florida reveals both higher J_s (Krauss et al. 2015a) and lower J_s (Krauss et al. 2007) relative to *R. mangle*, suggesting a role for locally-imposed site constraints on species-specific water use. A congeneric, *Avicennia marina*, was found to be the most sensitive among three mangrove species to the influence of environmental change on J_s in China (Wu et al. 2024), yet the same species was able to maintain J_s commensurate with nearby rainforest trees (Becker et al. 1997). Our knowledge of J_s response by *L. racemosa* to specific refuge conditions and whether that species adds to stand water use (and GPP), or offsets water usage (and GPP) with +N or +P loading, was not locally calibrated due to lack of individuals on plots and a lower representation of *L. racemosa* refuge-wide.

Box 1
Closure Analysis

Given that lateral C fluxes are estimated as an application of STrAP (Eq. 2, main text), we were able to compare STrAP's export portfolios versus an alternate approach, as follows,

$$\beta = NPP - \sum_{i=1}^{n}(Component_i) \qquad [S1]$$

Where β is basically an estimate of *partial* export, and NPP is the value derived from STrAP. Components include stem (c.1), litter (c.2), and root (c.3) productivity; R_s (c.4) and R_p (c.5); and soil C burial (c.6) and SSCA (c.7). Note that Equation 2 and Equation S1 should match if all C losses were included in the latter; however, we were not able to include stem respiration, *R. mangle* prop root respiration, and methane flux in component summations.

Table S3 Comparison of net primary productivity (NPP) estimation (g C m^{-2} y^{-1}) using the S-to-GPP modeling described in the main text vs. summing the components of NPP as measured by independent studies (Faron 2021; Conrad 2022; Conrad et al. 2024).

		a<br>STrAP export[a]	b<br>Components export[b]	c<br>(a) - (b)
Basin	Control	1287	890	397
	+N	1033	674	359
	+P	666	417	249
Fringe	Control	1198	834	364
	+N	1339	920	419
	+P	461	51	410
**Mean**				**366.3 ± 25.5**

[a] Export as estimated from Soil C burial (empirical) minus STrAP-derived NEE

[b] STrAP-derived NPP minus components

STrAP model closure – By comparing two different approaches to estimating C export from the STrAP model and through summing the components of productivity to determine NPP (Eq. 2 vs. S1), we can partially evaluate our *S*-to-GPP approach as STrAP export minus β. Closure between the two approaches averaged 366 ± 26 g C m^{-2} y^{-1} but ranged from 249 g C m^{-2} y^{-1} (+P, Basin) to 419 g C m^{-2} y^{-1} (+N, Fringe) (Table S3). Perfect closure is not expected given that not all components of NPP were assessed during the estimation of β (see main text).

Incorporating radial depth changes of J_s into a tree remains a critical variable for scaling from point measurements of sap flow using TDP or heat pulse velocity techniques to tree water usage (Ford et al. 2004; Zhang et al. 2015). Phillips et al. (1996) and James et al. (2003) discovered acute differences in J_s by radial depth into trees. Since, this pattern has been found to be pervasive in most tree species and locations (e.g., Jiménez et al. 2000; Nadezhdina et al. 2002; Kumagai et al. 2005), including mangroves (Krauss et al. 1997; Zhao et al. 2018). Yet, small trees or saplings often have diffuse patterns of J_s (Delzon et al. 2004). We did assume that trees < 7 cm *dbh* had a diffuse pattern of water usage across the full sapwood profile, as has been documented in small *A. germinans* in coastal Louisiana (Krauss et al. 2014a). +P altered how *A. germinans* trees used water at deeper sapwood depths such that where this species had a higher relative basal area versus *R. mangle* and *L. racemosa*, *S* was higher. The physiological response of mangroves to +P at DDNWR was driven by WUE_i shifts in *R. mangle* and in J_s-by-radial depth shifts in *A. germinans*, increasing *S* for +P-treated plots (Figure 3, main text).

Forest-scale eco-physiological changes

Fluxes of CO_2 that contribute to forest ecosystem GPP are especially sensitive to fluctuations in air temperature and relative humidity that control diffusion gradients of both water and CO_2 (Baldocchi 2003; Shoemaker et al. 2022). The approach inherent to the STrAP model, which we consider an "inverse modeling approach," considers atmosphere diffusion gradients for water and the feedback that water limitations impose on gross CO_2 uptake by the mangrove canopy. Inverse modelling accounts for gross CO_2 uptake and represents all the C entering the system through vegetation, or GPP. Validation is partially provided in Table S3; however, several additional respiratory fluxes need to be assessed for full closure analysis (Box 1).

Variation in functional sapwood area among trees of the same species and maximum values of J_s used for determining water use potential by size class and species provide a unique mechanism to assess potential C balance vulnerability by environmental stress incurred through an eco-physiological mechanism. Along with triangulating potential mechanisms for GPP shifts, the benefit of using inverse modeling versus eddy covariance (EC) approaches include, among ameliorating major cost and staffing barriers, an appropriate scale (tenths of hectares) to determine how experimental manipulations might influence GPP and NEE over short time periods. The scale of EC investigation spans many hectares for appropriately placed towers over mangrove forests (Barr et al. 2010; Lu et al. 2017); however, EC measures might provide the best way to validate our inverse modeling approach.

Application and improvements to approaches applied at DDNWR

Our use of leaf-level WUE_i values is an important component to detail, and perhaps improved upon in the future. Water use efficiency can be presented at three useful scales: leaf, tree, and ecosystem. Liang et al. (2022) detailed the formulation of each but indicated some transference among application scale, which is encouraging. The differences among scale can be summarized primarily by accounting for diffusion gradients between intercellular and atmospheric vapor pressure at the leaf scale versus tree scale, and a shift to growth variable formulation at the stand scale (Liang et al. 2022). Linkages between J_s and vapor pressure deficit (*D*) are widely described (e.g., Wullschleger et al. 2001; Bovard et al. 2005; Tang et al. 2006; Oishi et al. 2008), and available to improve upon stand-scale understanding of water usage to CO_2 gained. For example, future usage of intrinsic water use efficiencies (i.e., photosynthetic assimilation divided by stomatal conductance) show promise, but as a trade-off, might be less sensitive to local environmental drivers, such as +N and +P amendment.

Linderson et al. (2012) described a scaling approach from leaf WUE_i to canopy water use efficiency within an 80-year-old temperate beech forest (*Fagus sylvatica*) in Denmark. Scaling from leaf WUE_i to canopy WUE was successful, but they provide the caveat that both *D*, as we used here, and PPFD, as we relied on previously (Krauss et al. 2015b), are important to use in cross-walking the two scales. Re-

examining Liang et al. (2022), pressure gradients between leaf and atmosphere are a critical driver that, through formulation is reduced as a primary influence at the canopy-scale, suggesting that the influence of light to stimulate net photosynthesis would be important, especially in seasonal environments where sunlight may vary widely. Light is more consistent at DDNWR over an annual cycle than in Denmark. However, PPFD explained up to 75% of the variation in J_s for a mangrove forest in Everglades National Park, Florida, with *D* lagging in explanation to only 49% of the variation in J_s (Krauss et al. 2015a). Indeed, the same modeling approach was used at DDNWR as for temperate forested wetlands. The use of PPFD versus *D* relates to the application chosen; hindcasting *S* over the year was a good application for PPFD (reduces hysteresis) and determining maximum and average J_s values was a good application for *D* (Krauss et al. 2015b). While our approach at DDNWR was heavily dependent on *D*, we do incorporate PPFD when scaling to GPP values after WUE_i transformations are applied. We would like to refine this approach by incorporating a metric less dependent on relative humidity (and thus *D*), such as the marginal water use of C gain, or λ (Liang et al. 2023), and applying water use efficiency to canopies along a range of *D* (wet-to-dry) and PPFD (warm temperate to tropical).

Refuge management

The current nutritional state of DDNWR's mangrove habitat may already be driving deterioration of the system. Unnoticed degradation is common in mangroves, as their stress tolerance serves as a barrier to identifying pressing issues that might be ameliorated by implementation of management action (Lewis et al. 2016). Yet, mangroves currently located in basin settings demonstrated an ability to build soil surface elevations in the presence of rising sea levels despite DDNWR's current state of P eutrophication (Conrad et al. 2024). This capacity was commensurate with nearby basin mangroves in southwest Florida with far lower soil nutrient concentrations (Cahoon and Lynch 1997); however, some of the capacity to build soil surface elevations at DDNWR is related to the deposition of litter, branches, and wood particles from deteriorating canopies into unconsolidated soils. Vulnerabilities from degradation were confirmed through our C flux assessments, despite a capacity of the mangroves to build surface elevations even if lagging relative sea-level rise (Conrad et al. 2024). Treatments of +P were determined to reduce GPP at DDNWR even though root volume expansion is stimulated in other mangrove sites when P is added (McKee et al. 2007). Root C biomass production ranged from 131 to 244 g C m^{-2} y^{-1} and did not differentiate consistently with either +N or +P, but was highest in fringe +P plots (Conrad 2022).

With the exception of Jessen et al. (2022), previous studies focusing on fertilization of mangroves with +N and +P focused on fertilizing short mangroves in oligotrophic environments and tracking growth and eco-physiological differences among fertilizer regimes, e.g., root growth, leaf sclerophylly. Those studies were fundamental to what we currently know about mangroves, particularly in the Caribbean region. The mangrove forests at DDNWR are generally tall (10-15 m height) and developed under more optimal nutrient conditions originally, but have since been pressed with additional N and P from south Florida's most unregulated river from Lake Okeechobee. We expected a +N response with the idea that the system might benefit from an improved N:P ratio and because Jessen et al. (2022) did not find a +P response on sites in Puerto Rico. We discovered quite the opposite at DDNWR.

In the absence of P reductions from upstream of DDNWR, opportunities to use strategic land acquisitions to prevent development and provide options for DDNWR managers to apply their R-A-D (Resist-Accept-Direct) strategy (Lynch et al. 2022) may be the only way forward to maintain the health of the mangroves. Without uplands to accommodate mangrove migration, "accept" may be the default strategy, and significant mangrove areas may be lost in the future, exacerbated by metabolic stimulation with additional P loading from the Caloosahatchee River. Increased regulation of input into the river would tend toward "resist". NEE is an important proxy underpinning coastal wetland habitat persistence, and was a very revealing variable in our experiments. Accordingly, a brackish marsh in coastal Louisiana was

losing more C than it was gaining, and even with some C import, the balance was not offsetting local rates of subsidence and that wetland was submerging rapidly (Krauss et al. 2016). While subsidence is relatively low in DDNWR's mangroves, eventual loss of C with continued P loading will exacerbate in time to permanent submergence.

References

Appleby PG and Oldfield F (1983) The assessment of ^{210}Pb data from sites with varying sediment accumulation rates. *Hydrobiologia* **103**, 29–35. https://doi.org/10.1007/BF00028424

Baldocchi DD (2003) Assessing the eddy covariance technique for evaluating carbon dioxide exchange rates of ecosystems: past, present and future. *Global Change Biology* **9**, 479–492. https://doi.org/10.1046/j.1365-2486.2003.00629

Barr JG, **Engel V**, **Fuentes JD**, **Zieman JC**, **O'Halloran TL**, **Smith TJ III**, **et al.** (2010) Controls on mangrove forest-atmosphere carbon dioxide exchanges in western Everglades National Park. *Journal of Geophysical Research Biogeosciences*, *115*, GO2020. https://doi.org/10.1029/2009JG001186

Becker P, **Asmat A**, **Mohamad J**, **Moksin M and Tyree MT** (1997) Sap flow rates of mangrove trees are not unusually low. *Trees Structure and Function* **11**, 432–435. https://doi.org/10.1007/s004680050104

Bovard BD, **Curtis PS**, **Vogel CS**, **Su H-B and Schmid HP** (2005) Environmental controls on sap flow in a northern hardwood forest. *Tree Physiology*, **25** 31–38. https://doi.org/10.1093/treephys/25.1.31

Cahoon DR and Lynch JC (1997) Vertical accretion and shallow subsidence in a mangrove forest of southwestern Florida, U.S.A. *Mangroves and Salt Marshes*, **1**, 173–186. https://doi.org/10.1023/A:1009904816246

Carvajal M, **Cooke DT and Clarkson DT** (1996) Responses of wheat plants to nutrient deprivation may involve the regulation of water-channel function. *Planta* **199**, 372–381. https://doi.org/10.1007/BF00195729

Chambers LG, **Steinmuller HE and Breithaupt JL** (2019) Toward a mechanistic understanding of "peat collapse" and its potential contribution to coastal wetland loss. *Ecology* **100**, e02720. https://doi.org/10.1002/ecy.2720

Clarkson DT, **Carvajal M**, **Henzler T**, **Waterhouse RN**, **Smyth AJ**, **Cooker DT**, **et al.** (2000) Root hydraulic conductance: diurnal aquaporin expression and the effects of nutrient stress. *Journal of Experimental Botany* **51**, 61–70. https://doi.org/10.1093/jexbot/51.342.61

Conrad JR (2022) The effects of nutrient inputs on surface elevation change processes in tidal mangrove forests. Boca Raton, Florida Atlantic University, Ph.D. dissertation.

Conrad JR, **Krauss KW**, **Benscoter BW**, **Feller IC**, **Cormier N** and **Johnson DJ** (2024) Eutrophication saturates surface elevation change potential in tidal mangrove forests. *Estuaries and Coasts* **47**, 1814–1827. https://doi.org/10.1007/s12237-024-01353-8

Cormier N, **Krauss KW**, **Demopoulos AWJ**, **Jessen BJ**, **McClain-Counts JP**, **From AS**, **et al.** (2022) Potential for carbon and nitrogen sequestration by restoring tidal connectivity and enhancing soil surface elevations in denuded and degraded south Florida mangrove ecosystems, in *Wetland Carbon and Environmental Management*, edited by Krauss, K.W., et al., pp. 143–158, Wiley, New Jersey. https://doi.org/10.1002/9781119639305.ch7

Dahl M, **Lavery PS**, **Mazarrasa I**, **Samper-Villarreal J**, **Adame MF**, **Crooks S**, **et al.** (2025) Recommendations for strengthening blue carbon science. *One Earth* **8**, 101175. https://doi.org/10.1016/j.oneear.2025.101175

Delzon S, **Sartore M**, **Granier A and Loustau D** (2004) Radial profiles of sap flow with increasing tree size in maritime pine. *Tree Physiology* **24**, 1285–1293. https://doi.org/10.1093/treephys/24.11.1285

Demopoulos AWJ, **Bourque JR**, **McClain-Counts JP**, **Cormier N and Krauss KW** (2024) Benthic community metrics track hydrologically stressed mangrove systems. *Diversity* **16**, 659. https://doi.org/10.3390/d16110659

Drexler JZ (2019) Dry weight, volume and % organic carbon in mangrove sediment cores collected in September 2018 in J.N. Ding Darling National Wildlife Refuge, Sanibel Island, Florida, United States: *U.S. Geological Survey Data Release*. https://doi.org/10.5066/P9CFK79S

Drexler JZ, **Fuller CC**, **Orlando J**, **Salas A**, **Wurster FC and Duberstein JA** (2017) Estimation and uncertainty of recent carbon accumulation and vertical accretion in drained and undrained forested peatlands of the southeastern USA. *Journal of Geophysical Research Biogeosciences* **122**, 2563–2579. https://doi.org/10.1002/2017JG003950

Drexler JZ, **Orlando J and Archfield S** (2018) The approaching obsolescence of ^{137}Cs dating of wetland soils in North America. *Quaternary Science Reviews* **199**, 83–96. https://doi.org/10.1016/j.quascirev.2018.08.028

Faron NT (2021) The impact of nutrient loading on the soil and root respiration rates of Florida mangroves. Boca Raton, Florida Atlantic University, M.S. thesis.

Feller IC (1995) Effects of nutrient enrichment on growth and herbivory of dwarf red mangrove (*Rhizophora mangle*). *Ecological Monographs* **65**, 477–505. https://doi.org/10.2307/2963499

Feller IC, **McKee KL**, **Wingham DF and O'Neill JP** (2002) Nitrogen vs. phosphorus limitation across an ecotonal gradient in a mangrove forest. *Biogeochemistry* **62**, 145–175. https://doi.org/10.1023/A:1021166010892

Feller IC, **Wigham DF**, **McKee KL and Lovelock CE** (2003) Nitrogen limitation of growth and nutrient dynamics in a disturbed mangrove forest, Indian River Lagoon, Florida. *Oecologia* **134**, 405–414. https://doi.org/10.1007/s00442-002-1117-z

Ford CR, **McGuire MA**, **Mitchell RJ and Teskey RO** (2004) Assessing variation in the radial profile of sap flux density in Pinus species and its effect on daily water use. *Tree Physiology* **24**, 241–249. https://doi.org/10.1093/treephys/24.3.241

Granier A (1987) Evaluation of transpiration in a Douglas-fir stand by means of sap flow measurements. *Tree Physiology* **3**, 309–320. https://doi.org/10.1093/treephys/3.4.309

Holm GO Jr, **Perez BP**, **McWhorter DE**, **Krauss KW**, **Johnson DJ**, **Raynie RC**, **et al.** (2016) Ecosystem level methane fluxes from tidal freshwater and brackish marshes of the Mississippi River Delta: implications for coastal wetland carbon projects. *Wetlands* **36**, 401–413. https://doi.org/10.1007/s13157-016-0746-7

James SA, **Meinzer FC**, **Goldstein G**, **Woodruff D**, **Jones T**, **Restom T**, **et al.** (2003) Axial and radial water transport and internal water storage in tropical forest canopy trees. *Oecologia* **134**, 37–45. https://doi.org/10.1007/s00442-002-1080-8

Jessen BJ, **Oviatt CA**, **Rossi R**, **Duball C**, **Wignand C**, **Johnson DS**, **et al.** (2021) Decomposition of mangrove litter under experimental nutrient loading in a fringe *Rhizophora mangle* (L.) forest. *Estuarine, Coastal and Shelf Science* **248**, 106981. https://doi.org/10.1016/j.ecss.2020.106981

Jiménez MS, **Nadezhdina N**, **Čermák J and Morales D** (2000) Radial variation in sap flow in five laurel forest tree species in Tenerife, Canary Islands. *Tree Physiology* **20**, 1149–1156. https://doi.org/10.1093/treephys/20.17.1149

Krauss KW (2004) Growth, photosynthetic, and water use characteristics of south Florida mangrove vegetation in response to varying hydroperiod. Lafayette, University of Louisiana at Lafayette, Ph.D. dissertation.

Krauss KW and Osland MJ (2020) Tropical cyclones and the organization of mangrove forests: a review. *Annals of Botany* **125**, 213–234. https://doi.org/10.1093/aob/mcz161

Krauss KW, **Young PJ**, **Chambers JL**, **Doyle TW and Twilley RR** (2007) Sap flow characteristics of neotropical mangroves in flooded and drained soils. *Tree Physiology* **27**, 775–783. https://doi.org/10.1093/treephys/27.5.775

Krauss KW, **McKee KL and Hester MW** (2014a) Water use characteristics of black mangrove (*Avicennia germinans*) communities along an ecotone with marsh at a northern geographical limit. *Ecohydrology* **7**, 354–365. https://doi.org/10.1002/eco.1353

Krauss KW, **McKee KL**, **Lovelock CE**, **Cahoon DR**, **Saintilan N**, **Reef R**, **et al.** (2014b) How mangrove forests adjust to rising sea level. *New Phytologist* **202**, 19–34. https://doi.org/10.1111/nph.12605

Krauss KW, **Barr JG**, **Engel V**, **Fuentes JD and Wang H** (2015a) Approximations of stand water use versus evapotranspiration from three mangrove forests in southwest Florida, USA. *Agricultural and Forest Meteorology* **213**, 291–303. https://doi.org/10.1016/j.agrformet.2014.11.014

Krauss KW, **Duberstein JA and Conner WH** (2015b) Assessing stand water use in four coastal wetland forests using sapflow techniques: annual estimates, errors and associated uncertainties. *Hydrological Processes* **29**, 112–127. https://doi.org/10.1002/hyp.10130

Krauss KW, **Holm GO Jr**, **Perez BC**, **McWhorter DE**, **Cormier N**, **Moss RF**, **et al.** (2016) Component greenhouse gas fluxes and radiative balance from two deltaic marshes in Louisiana: pairing chamber techniques and eddy covariance. *Journal of Geophysical Research Biogeosciences **121***, 1503–1521. https://doi.org/10.1002/2015JG003224

Krauss KW, **Demopoulos AWJ**, **Cormier N**, **From AS**, **McClain-Counts JP and Lewis RR III** (2018) Ghost forests of Marco Island: mangrove mortality driven by belowground soil structural shifts during tidal hydrologic alteration. *Estuarine, Coastal and Shelf Science* **212**, 51–62. https://doi.org/10.1016/j.ecss.2018.06.026

Krauss KW, **Whelan KRT**, **Kennedy JP**, **Friess DA**, **Rogers CS**, **Stewart HA**, **et al.** (2023) Framework for facilitating mangrove recovery after hurricanes on Caribbean islands. *Restoration Ecology* **31**, e13885. https://doi.org/10.1111/rec.13885

Kumagai T, **Aoki S**, **Nagasawa H**, **Mabuchi T**, **Kubota K**, **Inoue S**, **et al.** (2005) Effects of tree-to-tree and radial variations on sap flow estimates of transpiration in Japanese cedar. *Agricultural and Forest Meteorology* **135**, 110–116. https://doi.org/10.1016/j.agrformet.2005.11.007

Lewis RR III, **Milbrandt EC**, **Brown B**, **Krauss KW**, **Rovai AS**, **Beever JW III**, **et al.** (2016) Stress in mangrove forests: early detection and preemptive rehabilitation are essential for future successful worldwide mangrove forest management. *Marine Pollution Bulletin **109***, 764–771. https://doi.org/10.1016/j.marpolbul.2016.03.006

Liang J, **Farquhar GD and Ball MC** (2022) Water use efficiency in mangroves: conservation of water use efficiency determined by stomatal behavior across leaves, plants, and forests. *Advances in Botanical Research* **103**, 43–59. https://doi.org/10.1016/bs.abr.2022.02.017

Liang J, **Krauss KW**, **Finnigan J**, **Stuart-Williams H**, **Farquhar GD and Ball MC** (2023) Linking water use efficiency with water use strategy from leaves to communities. *New Phytologist* **240**, 1735–1742. https://doi.org/10.1111/nph.19308

Linderson M-L, **Mikkelsen TN**, **Ibrom A**, **Lindroth A**, **Ro-Poulsen H and Pilegaard K** (2012) Up-scaling of water use efficiency from leaf to canopy as based on leaf gas exchange relationships and the modeled in-canopy light distribution. *Agricultural and Forest Meteorology* **152**, 201–211. https://doi.org/10.1016/j.agrformet.2011.09.019

Lovelock CE, **Ball MC**, **Martin KC and Feller IC** (2009) Nutrient enrichment increases mortality of mangroves. *PLoS ONE* **4**, e5600. https://doi.org/10.1371/journal.pone.0005600

Lovelock CE, **Adame MF**, **Bennion V**, **Hayes M**, **O'Mara J**, **Reef R**, **et al.** (2014a) Contemporary rates of carbon sequestration through vertical accretion vertical accretion of sediments in mangrove forests and saltmarshes of south east Queensland, Australia. *Estuaries and Coasts* **37**, 763–771. https://doi.org/10.1007/s12237-013-9702-4

Lovelock CE, **Feller IC**, **Reef R and Ruess RW** (2014b) Variable effects of nutrient enrichment on soil respiration in mangrove forests. *Plant and Soil* **79**, 135–148. https://doi.org/10.1007/s11104-014-2036-6

Lovelock CE, **Krauss KW**, **Osland MJ**, **Reef R and Ball MC** (2016) The physiology of mangrove trees with changing climate, in *Tropical Tree Physiology*, edited by Goldstein, G. & Santiago, L. S., pp. 149–179, Springer. https://doi.org/10.1007/978-3-319-27422-5_7

Lu W, **Xiao J**, **Liu F**, **Zhang Y**, **Liu C and Lin G** (2017) Contrasting ecosystem CO_2 fluxes of inland and coastal wetlands: a meta-analysis of eddy covariance data. *Global Change Biology* **23**, 1180–1198. https://doi.org/10.1111/gcb.13424

Lynch AJ, **Thompson LM**, **Morton JM**, **Beever EA**, **Clifford M**, **Limpinsel D**, **et al.** (2022) RAD adaptive management for transforming ecosystems. *BioScience* **72**, 45–56. https://doi.org/10.1093/biosci/biab091

McKee KL, **Feller IC**, **Popp M and Wanek W** (2002) Mangrove isotopic fractionation (δ^{15}N and δ^{13}C) across a nitrogen versus phosphorus limitation gradient. *Ecology **83***, 1065–1075. https://doi.org/10.1890/0012-9658(2002)083[1065:MINACF]2.0.CO;2

McKee KL, **Cahoon DR and Feller IC** (2007) Caribbean mangroves adjust to rising sea level through biotic controls on change in soil elevation. *Global Ecology and Biogeography* **16**, 545–556. https://doi.org/10.1111/j.1466-8238.2007.00317.x

Meyers JM, **Langtimm CA**, **Smith TJ III and Pednault-Willett K** (2006) Wildlife and habitat damage assessment from Hurricane Charley: recommendations for recovery of the J.N. "Ding" Darling National Wildlife Refuge Complex. *U.S. Geological Survey Open File Report*, *2006-1126*, Reston, Virginia, USA.

Milbrandt EC, **Greenawalt-Boswell JM**, **Sokoloff PD and Bartone SA** (2006) Impact and response of southwest Florida mangroves to the 2004 hurricane season. *Estuaries and Coasts* **29**, 979–984. https://doi.org/10.1007/BF02798659

Miller H (2022) Water use and nutrient retention in fertilized black and red mangroves in southwest Florida. Clemson, Clemson University, M.S. thesis.

Nadezhdina N, **Čermák J and Ceulemans R** (2002) Radial patterns of sap flow in woody stems of dominant and understory species: scaling errors associated with positioning of sensors. *Tree Physiology* **22**, 907–918. https://doi.org/10.1093/treephys/22.13.907

Noe GB, **Hupp CR**, **Bernhardt CE and Krauss KW** (2016) Contemporary deposition and long-term accumulation of sediment and nutrients by tidal freshwater forested wetlands impacted by sea level rise. *Estuaries and Coasts* **39**, 1006–1019. https://doi.org/10.1007/s12237-016-0066-4

Oishi AC, **Oren R and Stoy PC** (2008) Estimating components of forest evapotranspiration: a footprint approach for scaling sap flux measurements. *Agricultural and Forest Meteorology* **148**, 1719–1732. https://doi.org/10.1016/j.agrformet.2008.06.013

Pezeshki SR, **DeLaune RD and Patrick WH Jr** (1990) Differential response of selected mangroves to soil flooding and salinity: gas exchange and biomass partitioning. *Canadian Journal of Forest Research* **20**, 869–874. https://doi.org/10.1139/x90-116

Phillips N, **Oren R and Zimmermann R** (1996) Radial patterns of xylem sap flow in non-, diffuse-and ring-porous tree species. *Plant, Cell & Environment* **19**, 983–990. https://doi.org/10.1111/j.1365-3040.1996.tb00463.x

Poffenbarger HJ, **Needelman BA and Megonigal JP** (2011) Salinity influence on methane emissions from tidal marshes. *Wetlands* **31**, 831–842. https://doi.org/10.1007/s13157-011-0197-0

Saintilan N, **Khan NS**, **Ashe E**, **Kelleway JJ**, **Rogers K**, **Woodroffe CD, et al.** (2020) Thresholds of mangrove survival under rapid sea level rise. *Science* **368**, 118–1121. https://doi.org/10.1126/science.aba2656

Shoemaker WB, **Anderson FE**, **Sirianni MJ and Daniels A (**2022) Carbon fluxes and potential soil accumulation within Greater Everglades cypress and pine forested wetlands, in *Wetland Carbon and Environmental Management*, edited by Krauss, K. W., et al., pp. 371–384, Wiley, New Jersey, Wiley. https://doi.org/10.1002/9781119639305.ch20

Smith DM and Allen SJ (1996) Measurement of sap flow in plant stems. *Journal of Experimental Botany* **47**, 1833–1844. https://doi.org/10.1093/jxb/47.12.1833

Snedaker SC (1995) Mangroves and climate change in the Florida and Caribbean region: scenarios and hypotheses. *Hydrobiologia* **295**, 43–49. https://doi.org/10.1007/BF00029109

Tang J, **Bolstad PV**, **Ewers BE**, **Desai AR**, **Davis KJ and Carey EV** (2006) Sap flux-upscaled canopy transpiration, stomatal conductance, and water use efficiency in an old growth forest in the Great Lakes region of the United States. *Journal of Geophysical Research Biogeosciences* **111**, GO2009. https://doi.org/10.1029/2005JG000083

Troxler TG, **Barr JG**, **Fuentes JD**, **Engel V**, **Anderson G**, **Sanchez C**, **et al.** (2015) Component-specific dynamics of riverine mangrove CO_2 efflux in the Florida coastal Everglades. *Agricultural and Forest Meteorology* **213**, 273–282. https://doi.org/10.1016/j.agrformet.2014.12.012

Wang H, **Krauss KW**, **Noe GB**, **Dai Z and Trettin CC** (2023) Soil salinity and water level interact to generate tipping points in low salinity tidal wetlands responding to climate change. *Estuaries and Coasts* **46**, 1808–1828. https://doi.org/10.1007/s12237-023-01243-5

Wu S, **Gu** X, **Peng X and Chen L** (2024) Comparative analysis of water-use strategies in three subtropical mangrove species: a study of sap flow and gas exchange monitoring. *Tree Physiology* **44**, tpae102. https://doi.org/10.1093/treephys/tpae102

Wullschleger SD, **Hanson PJ and Todd DE** (2001) Transpiration from a multi-species deciduous forest as estimated by xylem sap flow techniques. *Forest Ecology and Management* **143,** 205–213. https://doi.org/10.1016/S0378-1127(00)00518-1

Zhang J-G, **He Q-Y**, **Shi W-Y**, **Otsuki K**, **Yamanaka N and Du S** (2015) Radial variations in xylem sap flow and their effect on whole-tree water use estimates. *Hydrological Processes* **29**, 4993–5002. https://doi.org/10.1002/hyp.10465

Zhao H, **Yang S**, **Guo X**, **Peng C**, **Gu X**, **Deng C**, **et al.** (2018) Anatomical explanations for acute depressions in radial patterns of axial sap flow in two diffuse-porous mangrove species: implications for water use. *Tree Physiolog*y **38**, 276–286. https://doi.org/10.1093/treephys/tpx172
